# Discovering Key Sub-Trajectories to Explain Traffic Prediction

**DOI:** 10.3390/s23010130

**Published:** 2022-12-23

**Authors:** Hongjun Wang, Zipei Fan, Jiyuan Chen, Lingyu Zhang, Xuan Song

**Affiliations:** 1 Department of Computer Science and Engineering, Southern University of Science and Technology, Shenzhen 518055, China; 2 Center for Spatial Information Science, The University of Tokyo, 4 Chome-6-1 Komaba, Meguro City, Tokyo 153-8505, Japan

**Keywords:** submodular, neural networks, explainable, Trajectory

## Abstract

Flow prediction has attracted extensive research attention; however, achieving reliable efficiency and interpretability from a unified model remains a challenging problem. In the literature, the Shapley method offers interpretable and explanatory insights for a unified framework for interpreting predictions. Nevertheless, using the Shapley value directly in traffic prediction results in certain issues. On the one hand, the correlation of positive and negative regions of fine-grained interpretation areas is difficult to understand. On the other hand, the *Shapley* method is an NP-hard problem with numerous possibilities for grid-based interpretation. Therefore, in this paper, we propose Trajectory Shapley, an approximate Shapley approach that functions by decomposing a flow tensor input with a multitude of trajectories and outputting the trajectories’ Shapley values in a specific region. However, the appearance of the trajectory is often random, leading to instability in interpreting results. Therefore, we propose a feature-based submodular algorithm to summarize the representative Shapley patterns. The summarization method can quickly generate the summary of Shapley distributions on overall trajectories so that users can understand the mechanisms of the deep model. Experimental results show that our algorithm can find multiple traffic trends from the different arterial roads and their Shapley distributions. Our approach was tested on real-world taxi trajectory datasets and exceeded explainable baseline models.

## 1. Introduction

With the development of wireless communication and location acquisition, people can easily acquire their location by using a smartphone with the Global Positioning System (GPS), which has resulted in a massive amount of fragmentary spatio-temporal (ST) data [1]. Therefore, taking full advantage of using such mobile data is key to meeting human mobility demands. In recent years, many researchers have studied ST data, such as crowd flow and traffic flow. Deep ST neural networks (e.g., ST-resnet [2], DeepST [3], DMVST-Net [4], STDN [5]) have demonstrated that deep networks can take maximum advantage of ST data for prediction.

Although those approaches predict future traffic flow with high accuracy, they are based on deep learning and involve stacked nonlinear operations, which are unexplainable and impede their deployment in cities. To understand black box systems, in recent years, great achievements have been made in convolutional neural network (CNN) visualization and interpretation. Saliency maps [6], which are a gradient-based method, back-propagate through the entire model from the output to the input, and exhibits a correlation score between each grid square of the input and output. An integrated gradient [7] analyzes a wide range of output and solves the problem of gradient saturation. Smooth gradient [8] removes noise during visualization.

In the meantime, there are various existing methods to interpret neural networks by extracting features in the field of image recognition, due to the high dimensions of the pixel space. Lime [9] and kernel SHAP [10] combine image segmentation and the transformation into superpixels, explaining each superpixel through ablation. Time2graph [11] extracts time-aware shapelets [12] using a two-level timing factor. By extracting the key timing signal, Time2graph constructs the shapelet evolution graph and successfully detects abnormal time series. Activation maximization [6] finds the input pattern that maximizes the activation value of a given hidden layer and uses the hidden layer to extract features. Although the above methods successfully explain the relevant domain model, they may be unfit for crowd prediction due to the different definitions of feature space. The crowd flow tensor *G* is defined by the summation of all independent trajectories (see Defination 1). The input for crowd prediction is additive (see Defination 2), which differs from other domains. In this paper, we intend to combine trajectories with the Shapley method and produce a relevant Shapley value for each trajectory. The Shapley value was first proposed by Shapley in the field of game theory and has recently been applied to explain neural networks [10]. The Shapley method is NP-hard, meaning that it is impractical to exhaust all possible resources. Its key feature is to separate *G* into multiple trajectories and explain how each trajectory performs independently. This solution converts the flow tensor into the result of the addition of multiple tracks, which can reduce the computational complexity from $O(2^{d\times H\times W})$ to $O(2^{N})$, where *d* is the history time slots, *H* is the height of the flow tensor, *W* is the width of the flow tensor, and *N* is the number of trajectories. However, despite reducing the solution space significantly, the trajectory space remains immense because, in real life, there are millions of cars on the road.

**Figure 1:**
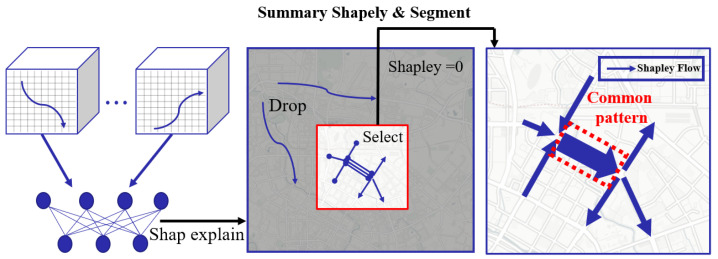
Overview of the proposed method. In this paper, we aim to quantify the significance of the trajectory of the Shapley flow among a set of input trajectories and discover the key Shapley flow forming a common pattern.

To address the above-mentioned challenges, in this paper, we combine the Shapley method with the trajectory tensor and propose a novel approach called Trajectory Shapley, which can compute approximate Shapley trajectories with time complexity O(N). Moreover, to solve the problem of the chaotic distribution of trajectories and to find the pattern of explainable common trajectories, we need to divide the trajectory into many small sub-trajectories because the long-distance trajectory pattern is random and difficult to explore. Sub-trajectories of the trajectory helps us to eliminate the unimportant and redundant fragments. Then, we use the submodular method to find *K* representative trajectories. Each representative trajectory represents a set of trajectories and a Shapley distribution. Our goal is for the distribution of different subsets to be as scattered as possible, to prove that we have found representative trajectories. The trajectory selection example shown in Figure 1.

Our contributions to the field are as follows:

(1) We propose Trajectory Shapley, a method that can effectively extract features from in–out flow and interpret neural networks. As far as we know, we are the first to introduce the Shapley value into crowd prediction;
(2) In order to understand the pattern of trajectories from randomly distributed trajectories, we use submodules to discover key sub-trajectories that are representative of a certain distribution;
(3) We validate the effectiveness of our approach on two real-world public datasets. Experimental results show that our approach achieves notably better performance in the aspects of coverage and summarization.

## 2. Architecture

**Figure 2:**
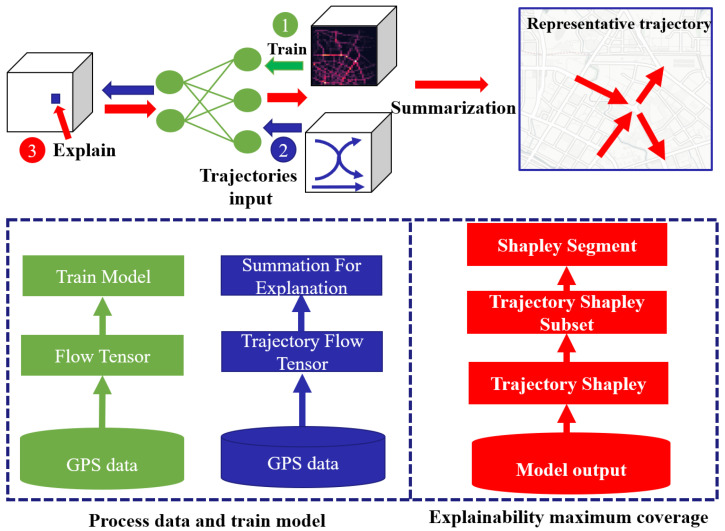
Framework for discovering key representative sub-trajectories.

Figure 2 shows the architecture of our explanatory process and the mining of the sub-trajectory correlation, which comprises two parts: data processing and model training, and maximum explainability coverage. The first part generates the flow tensor *G* and the trajectory flow tensor *T* in Defination 1 and Defination 2, respectively. The second part computes the trajectory Shapley values and finds the most representative *K* sub-trajectories through summarization.

**Data processing and model training:** Given multiple users’ GPS logs, we build two types of data: a flow tensor and a trajectory flow tensor. The flow tensor is generated to train a deep model, which is the same as in previous approaches. For explanation, we extract the trajectory flow tensor *T* from *G*. Note that the time and space complexity when using the input of *T* is *N* times greater than using *G*.

**Maximum explainability coverage:** There are four parts of this architecture: model output, Trajectory Shapley, Trajectory Shapley subset, and trajectory segment. The model output represents the deep model output with a summation of the trajectory flow tensor Equation (1). Trajectory Shapley is produced by grid-based Shapley values; see Section 4.1. The purpose of the Trajectory Shapley subset is to reduce the explanation space. We use the receptive field of the model to screen the subsets of trajectories to be explained. For details, see Section 4.2. The purpose of Shapley segmentation is to generate the solution space to discover the pattern of explainable common trajectories; see Section 4.3. The chaotic Trajectory Shapley distribution is summarized to provide a clear explanation for users; see Section 4.4.

## 3. Preliminaries

**Definition** **1** **(Inflow and outflow** [3]**).** *Let P be the set of trajectories in the $t^{th}$ time interval. For a grid of inflow and outflow matrices with i rows and j columns, the inflow $g_{t}^{in,i,j}$ and outflow $g_{t}^{out,i,j}$ of the crowds are defined as*

$$\begin{array}{cc} g_{t}^{in,i,j} & =\sum_{Tr\in P}\left|\left\{m>1|v_{m-1}\notin \left(i,j\right)\wedge v_{m}\in \left(i,j\right)\right\}\right|\\ g_{t}^{out,i,j} & =\sum_{Tr\in P}\left|\left\{m\geq 1|v_{m}\in \left(i,j\right)\wedge v_{m+1}\notin \left(i,j\right)\right\}\right| \end{array}$$

*where, $Tr:v_{1}\to v_{2}\to \cdots \to v_{\left|Tr\right|}$ is a single trajectory in P. $v_{m}\in \left(i,j\right)$ means that the trajectory $v_{m}$’s coordinates are in the region of $g_{t}^{i,j}$. |·| denotes the cardinality of a set.*

The inflow and outflow matrices are mixtures. Given an area to explain, it is difficult to attribute region contribution. Therefore, we extract the features from *G* following Defination 1. The equivalent definition for Defination 1 is

**Definition** **2** **(Trajectory flow splicing).***Let G be the flow matrix in the range of all time. Each trajectory can be split by a time interval as a tensor. Let* Ω *be the set of all trajectories; $T_{i}\in \Omega$ denotes a trajectory. $T_{i}^{in}$ and $T_{i}^{out}$ refer to a transfer presentation with the following constraint*

$$\begin{array}{cc} T_{r}^{in,i,j} & =\left|\left\{m>1|v_{m-1}\notin \left(i,j\right)\wedge v_{m}\in \left(i,j\right)\right\}\right|\\ T_{r}^{out,i,j} & =\left|\left\{m\geq 1|v_{m}\in \left(i,j\right)\wedge v_{m+1}\notin \left(i,j\right)\right\}\right| \end{array}$$

*Therefore, $G^{in}$ and $G^{out}$ are defined as*

(1) $$\begin{array}{cc} \; & G^{in}=\sum_{T_{r}^{in}\in \Omega }T_{r}^{in},\;G^{out}=\sum_{T_{r}^{out}\in \Omega }T_{r}^{out} \end{array}$$

**Figure 3:**
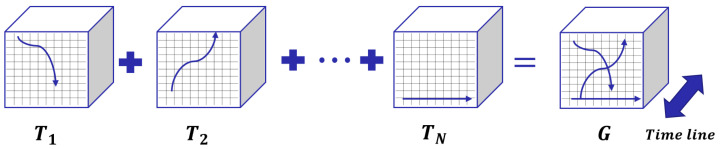
Schematic inflow and outflow matrices following Defination 2. For each trajectory, we calculate the inflow and outflow separately, and use the three-dimensional tensor $T_{i}^{in}$ and $T_{i}^{out}$ shapes as (timeline,x,y) to represent them; finally, we add all the tensors to obtain the final flow tensor *G*.

*The Figure 3 shows the aggregate process.*

**Definition** **3** **(Flow prediction).** *Given the historical observations $G_{t}=\left\{V_{t^{\prime }}|t^{\prime }\in \left[t-n+1,t\right]\right\}$, predict $Y_{t+1}$.*

(2) $$f:G_{t}\to Y_{t+1}$$

*where f is a neural network and n∈N denotes the length of the input timestamps, $V_{t}$ resides in $R^{1\times 2\times W\times H}$ as the one-frame inflow and outflow. W and H denote the region size.*

**Definition** **4** **(Shapley values** [13**]).** *The Shapley value is defined via the value function (val) of players in S and a feature value is its contribution to the payout, weighted, and summed over all possible feature value combinations:*

$$\begin{array}{c} \phi_{j}\left(val\right)=\sum_{S\subseteq \left\{x_{1},\ldots ,x_{p}\right\}\setminus \left\{x_{j}\right\}}\frac{|S|!(p-|S|-1)!}{p!}\left(val\left(S\cup \left\{x_{j}\right\}\right)-val\left(S\right)\right) \end{array}$$

*where S is a subset of the features used in the model, x is the vector of feature values of the instance to be explained, and p the number of features. ${\mathrm{val}}_{x}\left(S\right)$ is the prediction for feature values in set S. Ref.* [14] *showed that the Shapley value is the only reward with the following axioms.*

## 4. Trajectory Shapley

In this section, we present a novel algorithm for computing the Trajectory Shapley value. We name the proposed framework Trajectory Shapley, as it combines trajectory flow tensors and Shapley values.

### 4.1. Trajectory Shapley

While we extract the trajectory flow tensor and reduce the computational complexity from $O(2^{d\times H\times W})$ to $O(2^{N})$ within a certain time slot, there may be millions of trajectories in the city. The computational complexity is still large. Fortunately, Deep SHAP is a high-speed approximation algorithm for SHAP values in deep learning models that builds on a connection with Deep LIFT. The Trajectory Shapley value can be obtained with O(N) complexity. Following Defination 3, in crowd prediction, Deep SHAP can be formulated as

(3) $$\begin{array}{c} \phi \left(V_{t^{{}^{\prime }}}\right)=\left(Y_{t+1}-E\left[Y_{t+1}\right]\right)\times \frac{\partial (Y_{t+1}^{x,y})}{\partial V_{t^{{}^{\prime }}}},\;V_{t^{{}^{\prime }}}\in G_{t}, \end{array}$$

where $Y_{t+1}^{x,y}$ denotes the region coordinates (x,y) in the model output; $E\left[Y_{t+1}\right]$ is the expectation of output, which can be obtained approximately from the background samples. Therefore, we have the Shapley region $\phi (V_{t^{{}^{\prime }}})$. According to the chain rule, Definition 2, and Equation (3), we can obtain Trajectory Shapley $\phi (T_{i})$ with

(4) $$\begin{array}{cc} \phi (T_{i}) & =\left(Y_{t+1}-E\left[Y_{t+1}\right]\right)\times \frac{\partial (Y_{t+1}^{x,y})}{\partial T_{i}}\\ \; & =\left(Y_{t+1}-E\left[Y_{t+1}\right]\right))\times \frac{\partial (Y_{t+1}^{x,y})}{\partial V_{t^{{}^{\prime }}}}\times \frac{\partial V_{t^{{}^{\prime }}}}{\partial T_{i}}\\ \; & =\phi \left(V_{t^{{}^{\prime }}}\right)\times \frac{\partial V_{t^{{}^{\prime }}}}{\partial T_{i}} \end{array}$$

**Figure 4:**
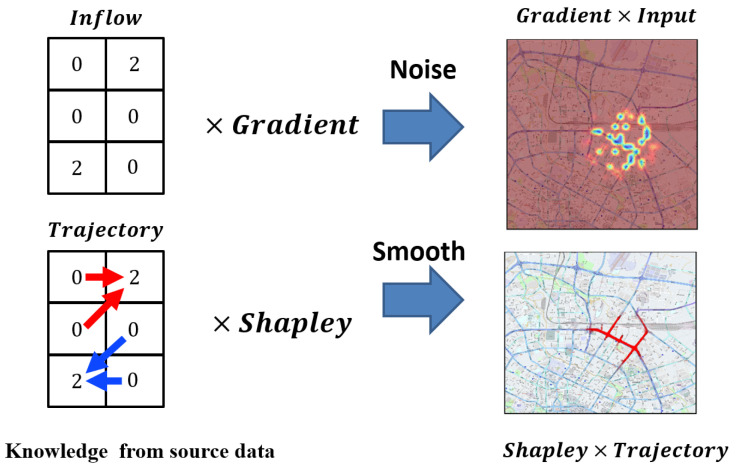
Input × Gradient vs. Trajectory × Shapley. The biggest difference between these is attribution. In traffic prediction tasks, using input × gradient rather than employing trajectories will result in the loss of attribution.

Therefore, the process of obtaining $\phi (T_{i})$ can be divided into two steps: (1) compute the region Shapley $\phi (V_{t^{{}^{\prime }}})$; (2) calculate Trajectory Shapley $\phi (T_{i})$ with $\phi (V_{t^{{}^{\prime }}})$. One of the benefits of using trajectories to explain traffic forecasting is attribution. According to Defination 1 and Figure 4, we know that there are four trajectories flowing into the two opposite grids, but we do not know where they come from. While using Gradient × Input can eliminate a significant amount of noise, such as the areas that no track pass through, some small particles are inevitably retained, because Gradient × Input removes the information on where the trajectory came from. On the contrary, for Trajectory × Shapley, by incorporating GPS logs, the prior information can help to attribute the trajectory and the results of interpretation are easy to understand. Here, we give the definition of the Shapley flow.

**Definition** **5** **(Shapley Flow).** *Given a trajectory T, the Shapley flow refers to the spatial and temporal contribution of the trajectory for a certain region in the model output.*

See Algorithm 1 for an overview of Trajectory Shapley.
**Algorithm 1** Trajectory Shapley**Input:**  Randomly selected background sample $S=\{G_{1},\cdots ,G_{\left|V\right|}\}$,  Explain region coordinate (x, y) in terms of grid,  Set trajectories $P=\{T_{1},\cdots ,T_{\left|N\right|}\}$ in *t* time slot,  Flow tensor $G_{t}$, pretrain model *f*. **Output:**
   1:Trajectory Shapley set *M*   2:Initialize $\phi (G_{t})=0$, M=∅   3:Generate output $Y_{t+1}=f\left(G_{t}\right)$   4:Obtain $E\left[Y_{t+1}\right]=\frac{1}{\left|V\right|}\sum_{G_{i}\in S}G_{i}$   5:Calculate $\phi (G_{t})$ using Equation (3)   6:**for all** trajectories $T_{i}$ in P **do**   7: Calculate $\phi (T_{i})$ using Equation (4)   8: Add $\phi (T_{i})$ to *M*   9:**end for** 10:**return** *M*


### 4.2. Maximum Explainability Coverage

It is difficult to analyze the Trajectory Shapley values obtained in Section 4.1 due to the large number of trajectories, chaotic distribution, (see Section 5.2.1), and the limitation on the number of roads in the spatial aspect, which leads to few paths being available for the trajectory. Furthermore, the trajectory flows are affected by the time of day, as in the morning and evening peaks, which provide the opportunity to discover special patterns. Therefore, many trajectories are redundant. To intuitively explain this to users, in this section, we discover key sub-trajectories to represent other trajectory signals.

### 4.3. Trajectory Segment

To discover the representativeness of the sub-trajectories, we need to discretize trajectories to find the common Shapley flow. For example, in Figure 1, three trajectories converge in three directions and then separate at the crossroads. In this kind of trajectory driving model, it is difficult to say which trajectory is representative. However, after segmentation, if we take each sub-trajectory as independent, we can easily find a common Shapley flow. Fortunately, the segment does not change the nature of the neural network due to additivity. The trajectory discrete equivalence theorem is as follows.

**Theorem** **1** **(Trajectory Discrete Equivalence).** *Take a neural network f and a set of trajectory tensors *Ω*, where trajectory tensor $T_{r}\in \Omega$. Each $T_{r}$ is segmented with multiple parts $t_{ri}$. We have the constraint $T_{r}=\sum_{i}t_{ri}$. Therefore, the neural network f has the same output as*

(5) $$\begin{array}{c} f\left(\sum_{r}T_{r}\right)=f\left(\sum_{r}\sum_{i}t_{ri}\right) \end{array}$$

The segmentation of trajectories can be obtained by executing the approximate trajectory partitioning algorithm [15]. We assume that the segmented trajectory is an independent trajectory. In other words, the trajectory in the submodular method is a set of line segments. According to Theorem 1, we can expand Trajectory Shapley Equation (4) to the sub-trajectories. After rerunning Trajectory Shapley, we obtain the Shapley value of each sub-trajectory. The two definitions of distance—perpendicular distance and angle distance—used in segments and submodules are as follows.

**Figure 5:**
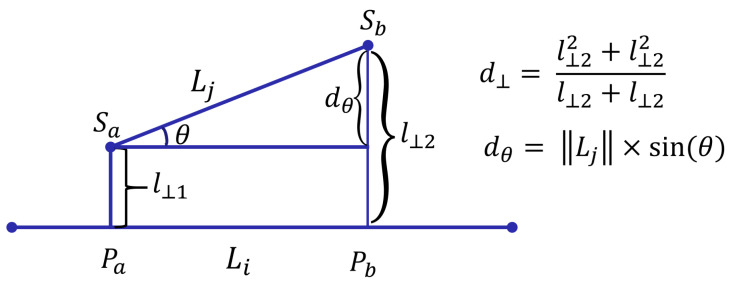
The distance function for line segments.

**Definition** **6** **(Perpendicular distance).** *Suppose the projection of the points $S_{a}$ and $S_{b}$ onto $L_{i}$ are $P_{a}$ and $P_{b},$ respectively. $l_{⊥1}$ is the Euclidean distance between $S_{a}$ and $P_{a}$; $l_{⊥2}$ is that between $S_{b}$ and $P_{b}$. The perpendicular distance is defined in Formula (6). Figure 5 shows the semantic of perpendicular distance.*

(6) $$\begin{array}{c} d_{⊥}\left(L_{i},L_{j}\right)=\frac{l_{⊥1}^{2}+l_{⊥2}^{2}}{l_{⊥1}+l_{⊥2}} \end{array}$$

**Definition** **7** **(Angle distance).** *The angle distance between $L_{i}$ and $L_{j}$ is defined in Formula (7). Here, $∥L_{j}∥$ is the length of $L_{j}$ and θ $\left(0^{\circ }\leq \theta \leq 180^{\circ }\right)$ is the smaller intersecting angle between $L_{i}$ and $L_{j}$. Figure 5 shows the semantic of angle distance.*

(7) $$\begin{array}{c} d_{\theta }\left(L_{i},L_{j}\right)=\left\{\begin{array}{cc} ∥L_{j}∥\times \sin\left(\theta \right), & \;\mathrm{if}\;0^{\circ }\leq \theta <90^{\circ }\\ ∥L_{j}∥, & \;\mathrm{if}\;90^{\circ }\leq \theta \leq 180^{\circ } \end{array}\right. \end{array}$$

### 4.4. Trajectory Shapley Maximum Coverage

Maximum coverage functions aim to maximize the number of features that have a non-zero element in at least one selected example; there is no marginal benefit to observing a variable in two examples. If each variable is thought to be an item in a set, and the data are a binary matrix where 1 indicates that the item is present in the example and 0 indicates that it is not, optimizing a maximum coverage function is a solution to the set coverage problem. These functions are useful when the space of variables is massive and each example only includes a small subset of them, which is a common situation when analyzing text data when the variables are words. The maximum coverage function is an instance of a feature-based function when the concave function is minimum. Maximum coverage is actually a special case of submodular maximization. Here, we have the definition of Trajectory Shapley maximum coverage.

**Definition** **8** **(Trajectory Shapley maximum coverage).** *Take a set of sub-trajectories *Ω* and their Shapley distribution $D_{total}$. Trajectory Shapley maximum coverage finds the K representativeness trajectories and K sub-distributions of*

$$\begin{array}{c} \min d_{t}\left(D_{total},D_{union}\right)-\sum_{i}^{K}\sum_{j}^{K}d_{t}\left(D_{i},D_{j}\right) \end{array}$$

*where $D_{i},D_{j}$ are the sub-Trajectory Shapley distributions and $D_{i},D_{j}\in R$. R is the set of  K sub-distributions. $D_{union}$ is the union of  R. $d_{t}$ is the distance function of two distributions.*

To achieve Trajectory Shapley maximum coverage in Defination 8, we divide the task into three parts: (1) similar Shapley flows should be as close as possible; (2) the sub-trajectories with large Shapley values should be chosen as the representative trajectories; (3) trajectories in same the cluster should be in similar directions and close to each other. For (1), we use the Euclidean distance to measure the segment Shapley ϕ(L) distance

(8) $$\begin{array}{c} d_{s}\left(\phi \left(L_{1}\right),\phi \left(L_{2}\right)\right)=||\phi \left(L_{1}\right)-\phi \left(L_{2}\right)|{|}^{2} \end{array}$$

For (2), on account of the large margin of Trajectory Shapley values, we apply the Sigmoid function to smooth them. Inspired by [16], we introduce a temperature parameter to adjust the sigmoid function $\delta \left(\phi \left(L_{i}\right),T\right)=\frac{1}{1+e^{-\frac{\left|\phi \left(L_{i}\right)\right|}{T}}}$, which modulates the weight of the Trajectory Shapley value and controls the distance between $L_{i}$ and $L_{j}$. We call *T* the Shapley weight. We can find that the smaller the Shapley weight *T*, the larger the distance of the pair of Shapley trajectories. By adjusting *T*, we can easily control the Trajectory Shapley distribution.

With Definitions 6 and 7, for (3), we use the perpendicular and angle distance to regularize the direction and distance of trajectories in the same cluster.

To sum up, we provide the asymmetric distance formula for a trajectory submodule of two sub-trajectories, $L_{i}$ and $L_{j}$:

(9) $$\begin{array}{cc} d_{L_{r}}\left(L_{1},L_{2}\right) & =\frac{\lambda_{\theta }d_{\theta }\left(L_{1},L_{2}\right)+\lambda_{⊥}d_{⊥}\left(L_{1},L_{2}\right)+\lambda_{s}d_{s}\left(\phi \left(L_{1}\right),\phi \left(L_{2}\right)\right)}{\delta (\phi \left(L_{r}\right),T)} \end{array}$$

where $L_{1},L_{2}\in L_{r}$. The selection matrix of maximum coverage can be obtained by setting threshold parameters ω. Through Theorem 2, the approximate solution of maximum coverage can be obtained by greedy maximum covering. Note that $\lambda_{\theta }$, $\lambda_{⊥}$, $\lambda_{s}$ control the weight of perpendicular distance, angle distance, and distribution distance, respectively.

**Theorem** **2.** *The greedy algorithm for monotone submodular maximization is a (1-1/e) approximation.*

**Proof.**  We use an argument similar to that used in Theorem 2. Let $S_{i}$ denote the set of elements chosen by the algorithm after *i* steps of the algorithm, and let $S^{*}$ be the set that maximizes f. Let $\ell_{i}$ be the difference between the value of these two sets, i.e., $\ell_{i}=f\left(S^{*}\right)-f\left(S_{i}\right).$ Our goal will again be to show that $\ell_{i}/k\leq \ell_{i}-\ell_{i+1}.$ Once we establish this, the proof becomes identical to that of Theorem 2.

To show this, let $K_{i}^{*}$ be the set of elements included in $S^{*}$ but not in *S* after *i* steps. Observe that since *f* is submodular, we have the following inequality:

$$\begin{array}{c} \sum_{j=1}^{\left|K_{i}^{*}\right|}f\left(S_{i}\cup \left\{y_{1},\ldots ,y_{j}\right\}\right)-f\left(S_{i}\cup \left\{y_{1},\ldots ,y_{j-1}\right\}\right)\\ \leq \sum_{j=1}^{\left|K_{i}^{*}\right|}f\left(S_{i}\cup \left\{y_{j}\right\}\right)-f\left(S_{i}\right) \end{array}$$

where $\left\{y_{1},\ldots ,y_{0}\right\}$ is defined as the empty set. Observe that the LHS of Equation (3) telescopes and therefore equals $f\left(S^{*}\cup S_{i}\right)-f\left(S_{i}\right).$ Since *f* is also monotone, we have $f\left(S_{i}\cup S^{*}\right)\geq f\left(S^{*}\right).$ Thus, Equation (3) implies that:

$$\begin{array}{c} \begin{array}{cc} \ell_{i}=f\left(S^{*}\right)-f\left(S_{i}\right) & \leq \sum_{j=1}^{\left|K_{i}^{*}\right|}f\left(S_{i}\cup \left\{y_{j}\right\}\right)-f\left(S_{i}\right)\\ \; & \leq \left|K_{i}^{*}\right|\max_{j\in K_{i}^{*}}\left\{f\left(S_{i}\cup \left\{y_{j}\right\}\right)-f\left(S_{i}\right)\right\}\\ \; & \leq k\left(\ell_{i}-\ell_{i+1}\right) \end{array} \end{array}$$

where (4) follows from the fact that the algorithm chooses the element that increases the value of *f* by the most and $K_{i}^{*}\leq k$ (the optimal solution can pick at most *k* elements). Thus, we have that $\ell_{i}/k\leq \ell_{i}-\ell_{i+1},$ as intended. As mentioned above, the rest of the proof is identical to that of Theorem 2.    □

See the formulation of the trajectory submodular framework in Algorithm 2.
**Algorithm 2** Trajectory Shapley maximum coverage**Input:**   The Trajectory Shapley set *M* calculated by Algorithm 1,   Trajectories set $P=\{T_{1},\cdots ,T_{\left|N\right|}\}$ in *t* time slot,   Submodular distance threshold ω. **Output:**   O={N sub-trajectories}
   1:Initialize the trajectories Shapley subsets $S=\left\{\phi \left(T_{i}\right)|\phi \left(T_{i}\right)\neq 0\wedge \phi \left(T_{i}\right)\in M\right\}$   2:Initialize the trajectories subsets $K=\left\{T_{i}|T_{i}\in P\wedge \phi \left(T_{i}\right)\in M\right\}$   3:Initialize segment set R={∅}, segment Shapley set S={∅}, segment distance matrix *D*   4:**for all** trajectory $T_{i}$ in P **do**   5: Run approximate trajectory partitioning algorithm for $T_{i}$   6: Add segment set *Q* to *R*   7: **for all** segment $L_{i}$ in *Q* **do**   8:  Rerun Algorithm 1 for $L_{i}$, adding $\phi (L_{i})$ to *S*   9: **end for** 10:**end for** 11:**for all** segment $L_{i}$ in *Q* **do** 12: **for all** segment $L_{j}$ in *Q* **do** 13:  Calculate $d_{L_{i}}\left(L_{i},L_{j}\right)$ and $d_{L_{j}}\left(L_{i},L_{j}\right)$ using Equation (9). 14:  Set $D\left(L_{i},L_{j}\right)=d_{L_{i}}\left(L_{i},L_{j}\right)$ and $D\left(L_{j},L_{i}\right)=d_{L_{j}}\left(L_{i},L_{j}\right)$ 15: **end for** 16:**end for** 17:Set $D=\left\{1|D(i,j)\;\leq \omega \right\}$ and $D=\left\{0|D(i,j)\;>\omega \right\}$ 18:Run greedy max cover algorithm and find *N* common patterns. 19:**return** *O*


## 5. Experiments

In this paper, we used a large-scale online taxi request dataset collected from DiDi Chuxing, which is one of the largest online car-hailing companies in China. One dataset contains taxi requests from 1 November 2016 to 30 format 2016 for the city of Chengdu. There are 38 × 36 regions in our data. The size of each region is 0.450 km × 0.450 km. Another dataset contains taxi requests from 1 October 2016 to 30 October 2016 for the city of Xi’an. There are 39 × 32 regions in our data. The size of each region is 0.450 km × 0.450 km. We used the Chengdu data from 1 November 2016 to 23 November 2016 for training (23 days), and the data from 24 November 2016 to 30 November 2016 (7 days) for testing. The Xi’an data from 1 October 2016 to 24 October 2016 was used for training (24 days), and the data from 25 October 2016 to 31 October 2016 (7 days) was used for testing. We used 10 min as the time interval.

### 5.1. Classic Prediction Methods for Comparison

We compared the proposed Trajectory Shapley model with three classic baselines, with these baselines trained on the Chengdu and Xi’an datasets:

• CNN: We used a basic deep learning predictor constructed with four CNN layers. The 4D tensor is represented by $\left(H,T,W,C\right)$. The CNN predictor utilizes four Conv layers to take the current observed *t*-step frames as input and predicts the next frame as output;
• ST-GCN [17]: For ST-GCN, we set the adjacency matrix to have the same receptive field as the CNN. The receptive field was set on the basis of a grid. It is regulated by the distance parameter ω. The three layer channels in the ST-Conv block were 64, 64, and 64, respectively. Both the graph convolution kernel size *K* and temporal convolution kernel size $K_{t}$ were set to 3 in the model.
• DNN: We straightened in–out flow grids into vectors and used them as the output of DNNs. We also erased the time information in DNNs and used five layers of a fully connected network. The feature size of each layer was T×W×C.

### 5.2. Case Study

#### 5.2.1. Case Study of Trajectory Shapley Visualization

**Figure 6:**
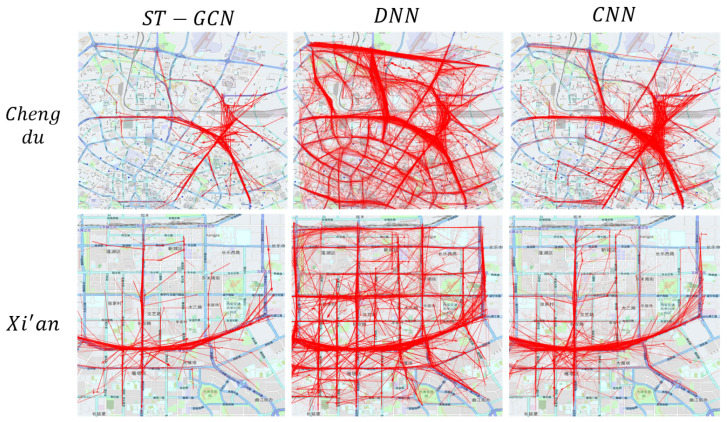
The simulation experiments of Trajectory Shapley in Chengdu and Xi’an with different classical prediction models.

The Figure 6 shows the performance of Trajectory Shapley with a CNN, ST−GCN, and DNN on the same region. We used transparency to represent the Shapley value of the trajectory. The time slot we chose was from 8:00 to 8:50 both for Xi’an and Chengdu. The area we selected was Xian’s overpass with the Chang’an interchange and Chang’an Road. In one day, there were 66 DiDi taxis passing by every 10 min. In Chengdu, we chose the intersection of the Second Ring Elevated Road and Fuqing Road. The speed here is fast and the traffic flow is large; it is the place with the largest traffic flow. We chose these areas because their flows are the greatest over the whole day in the two cities. The model pays more attention to this area due to the loss function, so the visualization area is representative. We can see that with the CNN and ST-GNN, the receptive fields of the models are limited by the depth of the models and the size of the kernel, so they are similar. However, with the DNN, perception is global because the DNN eliminates spatial information. The distribution of trajectories in each classical method is turbid and disordered; thus, we propose the summarization method to conclude the Shapley distribution.

#### 5.2.2. Case Study of Explainable Summarization

We use an example to explain the process of mining representative trajectories. In this experiment, we tested the morning and evening peaks of taxi driving in Chengdu, from 8:00 to 8:50 and from 18:00 to 18:50, respectively, on 1 November 2016. There are 12,971 and 10,871 tracks in these time periods, respectively. We chose the same place in Section 5.2.1 in Chengdu. Due to space limitations, we only display the CNN model results here.

##### Subset Trajectories

**Figure 7:**
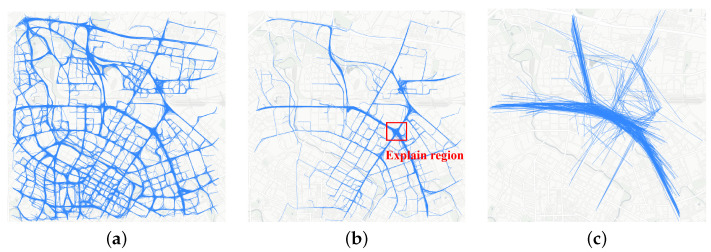
Finding a subset of interpretable trajectories. (**a**) City-wide trajectory. (**b**) Subset tracks and explanation region. (**c**) Sub-trajectory summarization set.

By calculating the Shapley value of each trajectory with O(N) time complexity, we can easily filter out a large number of irrelevant trajectories; then, we retain 533 trajectories in the morning case, as shown in Figure 7b.

##### Subsets Segment

We used the trajectory segment approach to segment 533 tracks in Figure 7b. Then, we recalculated the Shapley value of each line segment and filtered out the line segments with $\phi (L_{i})=0$. Finally, we obtained the sub-trajectory summarization set in Figure 7c; 487 segments were retained.

##### Trajectory Shapley Cover

**Figure 8:**
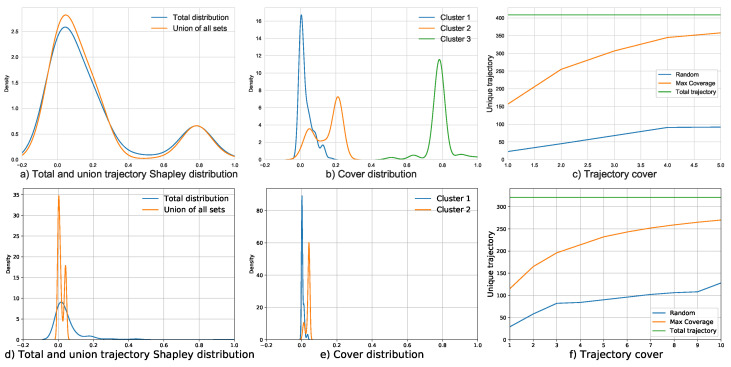
Coverage results of morning peak and evening peaks.

We used Formula (9) to compute the asymmetric distance for each sub-trajectory and set the 5% quantile as the distance coverage parameter ω to divide the coverage. The segment coverage matrix D∈{0,1}. Each row and column represents a segment; 1 means that the segments cover each other, 0 means that they are unrelated. Note that the segment coverage matrix is the input of summarization and the goal is to select *K* sets to cover all samples. The result of trajectory coverage is shown in the Figure 8. Figure 8c,f show the algorithm results for the morning and evening peaks, with the green line representing the whole set, the orange line representing the Trajectory Shapley maximum coverage algorithm, and the blue line representing the randomly selected set. It should be noted that there are several reasons why the coverage is not complete. (1) Our goal is to find the representative distribution and the common Shapley flow under this distribution; therefore, the coverage is as complete as possible. (2) Coverage is influenced by the covering parameter ω. (3) Even if we conduct segmentation, there are still some segments passing through different regions, such as in Clusters 1 and 2. Therefore, such segments are difficult to cover and their Shapley values are relatively random.

##### Common Shapley Flow

The common Shapley flow of a submodular cover describes the overall importance of the trajectory partitions that belong to the cluster. We need to extract quantitative information on the movement within a cluster such that domain experts can understand the movement in the trajectories. Thus, to gain full practical potential from trajectory clustering, a representative trajectory is required. Figure 8b,e show the distribution of the cluster. For the morning peak, we found three obvious patterns, while for the evening peak, we found two patterns. We can find that their distributions vary and try to cover different areas. In Figure 8a,d, we display the original Shapley trajectories and the union of cluster distribution. In the morning case, two distributions cover the main body, and one distribution covers the large Shapley value region. In the evening case, all distributions are focused on the main body region. Note that the distribution and coverage can be adjusted by five parameters, which are discussed in Section 5.3.

##### Result Analysis

**Figure 9:**
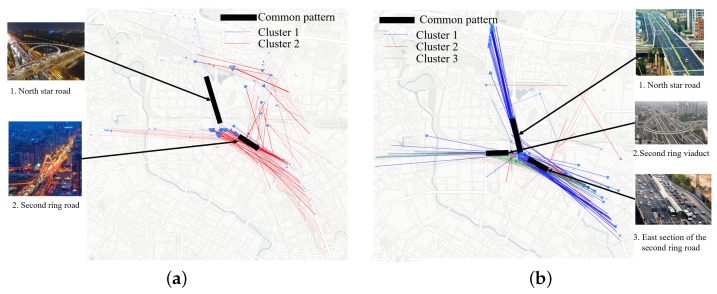
Comparison of the key Shapley flow and the trend of flows during morning and evening rush hours in Chengdu. (**a**) The pattern when evening peak. (**b**) The pattern when morning peak.

The results of spatial visualization are displayed in Figure 9. We use transparency to represent the Shapley value, which is the same as Figure 6. We display the trajectory directions to reflect the trend of traffic flow. We can see that since most of the people live in the suburbs, in the morning, the traffic flow mainly comes from North Star Road and then passes through Second Ring Road. This was successfully perceived by the neural network. Therefore, the distribution with the largest Shapley weight is the part with blue sub-trajectories. On the contrary, during evening rush hour, people move from the city to the suburbs. The second loop traffic is successfully mined by the model. We only show the first two classes because the Shapley value in the latter classes is too small. The reason why common the Shapley flow is short and near the interpretation region is that it is affected by perpendicular distance and weight. On one hand, if the common Shapley flow is very long, it is difficult to balance segments from all directions, which is mainly affected by the coverage. On the other hand, according to Equation (4), we know that the closer to the interpretation area, the greater the weight. Our algorithm gives priority to trajectories with large Shapley values.

### 5.3. Parameter Analysis

We examine the sensitivities of five important hyperparameters: the weight of perpendicular distance $\lambda_{\theta }$, angle distance $\lambda_{⊥}$, distribution distance $\lambda_{s}$, the coverage parameter ω, and the Shapley weight *T*. We use coverage $P_{cover}$ to reflect the ability of the algorithm to cover samples, which can be written as

(10) $$\begin{array}{c} P_{cover}=\frac{\cup U_{i}}{N}, \end{array}$$

where $U_{i}$ denotes the *i* sample cover set, *N* denotes the total number of samples, and cup denotes the union operation. Moreover, intuitively, the common patterns for each coverage should be distinguishable; otherwise, they should be merged into one block, so we use the distance of different distributions to measure the influence of parameters. Here, we introduce the Wasserstein distance

(11) $$\begin{array}{c} W\left(P_{r},P_{g}\right)=\underset{\gamma ∼\Pi \left(P_{r},P_{g}\right)}{\inf}E_{(x,y)∼\gamma }[∥x-y∥], \end{array}$$

where $\Pi \left(P_{r},P_{g}\right)$ is the set of all possible joint distributions that are combined for $P_{r}$ and $P_{g}$. For every possible joint distribution γ, we can sample from (x,y)∼γ to obtain real samples *x* and *y*. The expected value $E_{(x,y)∼\gamma }[∥x-y∥]$ of the sample with respect to the distance under the joint distribution γ can be calculated.

**Figure 10:**
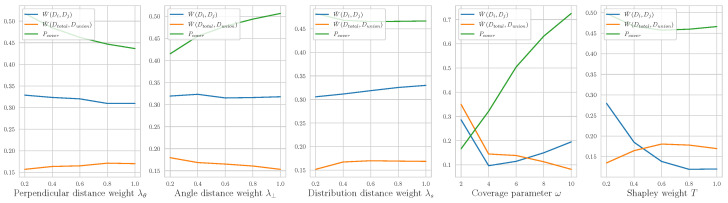
Parameter analysis.

We set the average distance between all samples as $\bar{W}\left(D_{i},D_{j}\right)$ and the average distance between the sample distribution and the union of the set distribution as $\bar{W}\left(D_{total},D_{union}\right)$. The range of $\lambda_{\theta }$, $\lambda_{⊥}$, $\lambda_{s}$, *T* ∈{0.2,0.4,0.6,0.8,1}, and ω∈{2,4,6,8,10}. From the results in Figure 10, we see that $\bar{W}\left(D_{total},D_{union}\right)$ is stable at all times except when changing the segment receptive field ω or the Shapley weight *T*. This shows the stationarity of the algorithm, which covers the overall distribution as much as possible. $\bar{W}\left(D_{total},D_{union}\right)$ have an inverse relationship with ω and *T*. This is intuitively reasonable because if the receptive field is too small, it will be indistinguishable. Moreover, enlarging *T* will eliminate the effects of Shapley values. If T=∞, the asymmetric distance will transform to symmetry. In all five cases, $\bar{W}\left(D_{i},D_{j}\right)$ remains at about 0.3. This shows that the distance between each class is relatively stable. ω should be large enough to cover as many samples as possible, which is reflected in $P_{cover}$. $P_{cover}$ is proportional to $\lambda_{⊥}$, inversely proportional with $\lambda_{\theta }$, and related to $\lambda_{s}$ and *T*. If the perpendicular distance $\lambda_{⊥}$ increases, there will be fewer segments. If the angle distance $\lambda_{\theta }$ increases, the algorithm will be more likely to select a similar angle. It is reasonable that changes of distribution distance $\lambda_{s}$ and *T* have little effect on the coverage because these are mainly used to adjust the clustering of the Shapley distribution.

## 6. Related Work

### 6.1. Urban Computing and Crowd Prediction

GPS data [18,19,20], social network data [21,22], and query data [23] have been extensively researched in recent years. Massive datasets have been published and relevant studies have demonstrated the potential of big data to solve the difficult problems in urban computing, for example, traffic jams [24], supply–demand [25], and energy consumption [26]. The classic review [27] summarizes the key challenges, general framework, and applications of urban computing. Many studies have proposed different methods for the task of crowd prediction, such as DCRNN [28], SRCNs [29], and multitask-net [30]. VLUC [31], PCRN [32], and PDB-ConvLSTM [33] use CNNs to process recent, near, and far data, respectively, and treat each timestamp as the equivalent convolution channel. STGCN [17], MRGCN [34], and ST-MGCN [34] fit a graph to the road structure and use convolution to learn temporal correlations. However, practical experiments are still lacking to explain how these models produce their results by learning the features from an input. Therefore, in this work, we propose a novel framework that focuses on dealing with mixed trajectory inputs. Secondly, our model attempts to summarize and attribute the Shapley value to trajectories.

### 6.2. Explainable Model

Linear models or basic decision trees are still widely used in many application that require a highly explainable model, even at the expense of a large compromise in accuracy. However, recent works with elaborately designed interpretation techniques [6,35] have demonstrated how neural networks obtain the mapping relation between input and output and have represented the decision-making process of neural networks. A general framework to achieve model-agnostic explanation is to visualize and understand the activation value produced by the neural network. Deconvolution [36] maps the features of the activation function back to the grid space to reveal what input patterns produce a particular output. Guided backpropagation [37] replaces the pooling operation with stride convolution, while ReLU backpropagation [38] prevents the backwards flow of negative gradients. Game theory can be used to calculate the importance of each feature [10]. However, these methods are not effective for crowd prediction, since crowd prediction maintains spatial and temporal patterns. Furthermore, the direct use of these methods in crowd prediction is difficult due to the lack of attribution.

### 6.3. Trajectory Cluster

Clustering similar trajectories to produce representative exemplars can be a powerful visualization tool to track the mobility of vehicles and humans. It has been investigated for many different applications, such as spatial databases [39,40], data mining [41], transportation [42], motion segmenting [43], and visualization [44]. Clustering is most often applied to spatial-only trajectories, with prior work on spatial-textual trajectory clustering being relatively rare. Trajectory clustering can be broadly divided into two categories: partition-based clustering [44,45,46] and density-based clustering [39,47,48]. Both partition- and density-based trajectory clustering require extensive similarity computations, with the only distinction being whether similarity is computed for whole trajectories or using only sub-trajectories. However, these methods have not been applied for mining representative model patterns.

## 7. Conclusions and Discussion

In this paper, we proposed a novel framework called Trajectory Shapley to explain spatial and temporal correlation in flow prediction. To capture common patterns for model prediction, we proposed the idea of summarizing Trajectory Shapely value distributions. We demonstrate the theory of Trajectory Shapley and show that our method produces a structural and continuous result that is easy to understand for users. We conducted experiments on two real-world public datasets from *DiDi*, using morning and evening rush hours as comparison experiments to test whether our submodule can successfully capture information in time and space. We demonstrated the effectiveness and interpretability of our proposed model, which can obtain the Trajectory Shapley value with time complexity O(N). In the future, we will try to explore more diverse patterns, such as flocking, gathering, swarming, or meeting [49], in explaining crowd prediction.

## Data Availability

This paper did not report any data.
